# Apoptotic and proliferative processes in the small intestine of rats with type 2 diabetes mellitus after metformin and propionic acid treatment

**DOI:** 10.3389/fphar.2024.1477793

**Published:** 2024-10-16

**Authors:** Larysa Natrus, Olha Lisakovska, Anton Smirnov, Yuliia Osadchuk, Yuliia Klys

**Affiliations:** ^1^ Department of Modern Technologies of Medical Diagnostics and Treatment, Bogomolets National Medical University, Kyiv, Ukraine; ^2^ Department of Biochemistry of Vitamins and Coenzymes, Palladin Institute of Biochemistry, Kyiv, Ukraine; ^3^ Department of Socio-Humanitarian and Biomedical Sciences, Kharkiv Institute of Medicine and Biomedical Sciences, Kharkiv, Ukraine

**Keywords:** propionic acid, metformin, type 2 diabetes mellitus, small intestine, diabetic intestinal complications, apoptosis, proliferation, mitochondria

## Abstract

**Background:**

Propionic acid (PA) is an intermediate product of metabolism of intestinal bacteria and may protect the intestinal barrier from disruption. The aim of the study was to investigate the apoptotic and proliferative processes in the small intestine (SI) of rats with type 2 diabetes mellitus (T2DM) on the background of metformin monotherapy and its combination with PA.

**Methods:**

Male Wistar rats were divided: 1) control; 2) T2DM (3-month high-fat diet followed by streptozotocin injection of 25 mg/kg of body weight); 3) T2DM + metformin (60 mg/kg, 14 days, orally); 4) T2DM + PA (60 mg/kg, 14 days, orally); 5) T2DM + PA + metformin. Western blotting, RT-PCR, and scanning electron microscopy were performed.

**Results:**

We observed profound changes in the SI of diabetic rats suggesting the disturbed intestinal homeostasis: impaired mitochondrial ultrastructure, increased cristae volume, and decreased content of proliferative marker Ki67 with almost unchanged proapoptotic caspase-3 and its p17 subunit levels. Metformin and PA monotherapies also led to an increased cristae volume, however, after their combination, a tendency to normalization of ultrastructure of mitochondria was observed. While there was a significant inhibition of proliferation in T2DM and, in greater extent, after metformin and PA monotherapies, differential influence on apoptosis in the SI was observed. While metformin inhibited apoptosis via Bax declining, PA mainly acted via caspase-3-dependent mechanism elevating its active p17 subunit.

**Conclusion:**

PA supplementation for the improvement of diabetes-induced gastrointestinal complications concurrently with metformin may be consider as a perspective supportive therapy. Data related to PA action on SI may be valuable during the development of new treatment strategies for diabetes-induced intestinal disturbances raised after metformin treatment.

## 1 Introduction

Global prevalence of diabetes is increasing rapidly, in particular, type 2 diabetes mellitus (T2DM) accounts for approximately 96% of the cases of diabetes (Ong et al., 2023). Individuals with T2DM are at an increased risk of the development of multiple complications, including gastrointestinal (GI) manifestations (Gotfried et al., 2017). The usual treatment for intestinal complications includes dietary modifications, prokinetic and antiemetic agents (Parkman et al., 2010). However, limited data are available on the specific gastrointestinal supporting therapy among diabetic patients. From a public health perspective, further studies to develop novel approaches for managing diabetic intestinal complications, especially on the background of common metformin anti-diabetic therapy, are critical.

Metformin has been known for about 100 years, and for more than 60 years it has been successfully applied as a glucose-lowering agent. Despite this drug is the first line of treatment in most patients who require oral antidiabetic therapy and is considered to be safe and effective, its molecular mechanisms of action still remain widely debated. Metformin is efficiently absorbed by the apical surface of enterocytes via specific transporters OCT1, PMAT, SERT and CHT, accounting for approximately 25%, 20%, 20%, and 15% of apical metformin transport, respectively (Han et al., 2015; McCreight et al., 2016). However, its transport through the basolateral surface of enterocytes is limited that may cause an accumulation of metformin in the wall of the intestine (Proctor et al., 2008; Bailey et al., 2008). Metformin concentration in the jejunum may reach 30–300 times higher than the plasma concentrations (Bailey et al., 2008). Altogether, these facts indicate that small intestine is an important site of metformin uptake.

Therefore, it is not surprising that metformin treatment is associated with GI side effects including diarrhea, abdominal pain, nausea and vomiting (Bouchoucha et al., 2011), in approximately 20%–30% of patients, leading to discontinuation of treatment in approximately 5% of patients (Dujic et al., 2015; Rena et al., 2017; Vancura et al., 2018). It has been shown that metformin significantly influences intestinal functioning via increasing lactate production, glucagon-like-peptide-1 level, cholesterol and bile acid pool, as well as changing intestinal microbiome (Graham et al., 2011; Dujic et al., 2015; McCreight et al., 2016; Rena et al., 2017). In turn, metformin-associated microbiome alteration may be a potential cause of drug intolerance. A crossover study showed that the treatment with metformin in combination with gastrointestinal microbiome modulator resulted in lowering fasting glucose levels that might reduce metformin-related GI adverse effects in patients with metformin GI intolerance and improve diabetes treatment (Burton et al., 2015). This study emphasizes the importance of the additional correction of T2DM- and metformin-related GI complications. Moreover, since T2DM treatment may take a long time, it is necessary to improve therapeutic strategies with a focus on the target organs including intestine.

Therefore, we consider it appropriate to study the molecular mechanisms of metformin action on the small intestine in combination with other substances on an adequate animal model in order to diminish diabetes-related GI complications and negative impact of metformin on GI tract. As an additional substance for GI supportive therapy, we consider sodium salt of propionic acid (PA). PA, one of the major short-chain fatty acids (SCFAs), is an intermediate product of the metabolism of intestinal bacteria. It is found in natural foods as well as widely used as a common food preservative. PA along with other SCFAs is one of the main energy sources for intestinal epithelial cells, and induces the expression of key genes involved in gluconeogenesis in enterocytes (Zhan et al., 2020). SCFAs are thought to stimulate intestinal barrier formation and protect the intestinal barrier from lipopolysaccharide-induced degradation by inhibiting the NLRP3 inflammasome and autophagy (Feng et al., 2018). Moreover, it was shown that gut microbiota-derived propionate reduces cancer cell proliferation in the liver (Bindels et al., 2012). However, underlying cellular/molecular underpinnings of how SCFAs can restore intestinal barrier dysfunction in physiological, and, moreover, in pathological conditions, are still uncovered. Moreover, a special attention on the possible propionate toxicity, especially in the context of an important role in the gut-brain axis functioning, should be paid, and it may be an interesting point considering a current spread of exogenous propionate-containing products for the treatment of neurodegenerative diseases and protection against neuroinflammation.

Thus, considering that current preventive and/or therapeutic options for gastrointestinal complications of T2DM and long-term metformin therapy may include not only dietary control, but supplementation of additional nutrients, our hypothesis is whether PA could exert possible protective effects on the diabetes-induced disturbances in small intestine on the background of metformin therapy. The main focus of the study was on apoptosis and proliferation since these processes represent crucial parts of the physiological tissue turnover, and the balance between them is important for maintenance of intestinal homeostasis. Therefore, the present study was designed to investigate the apoptotic and proliferative processes in the small intestine of rats with T2DM on the background of metformin monotherapy and its combination with propionic acid.

## 2 Materials and methods

### 2.1 Rat model of type 2 diabetes mellitus and experimental protocol

A total number of 60 adult male Wistar rats weighing 150.2 ± 9.5 g were used in the current study. Animals were housed in an environmentally controlled clean room (24°C ± 2°C, 65% ± 5% humidity), an alternating 12 h light/12 h dark cycle, and fed a standard rodent diet (Rezon-1, Ukraine) and water *ad libitum*. The experimental protocol was approved by the Bioethics Committee of the Bogomolets National Medical University (Protocol No 176 from 09/10/2023). All experimental procedures with animals were carried out in an accordance with the national and international guidelines concerning animal welfare: “European Convention for the protection of vertebrate animals used for experimental and other scientific purposes” (Strasbourg, 1986), “Bioethical expertise of preclinical and other scientific research conducted on animals” No. 3447-IV (Kyiv, 2006).

**FIGURE 1:**
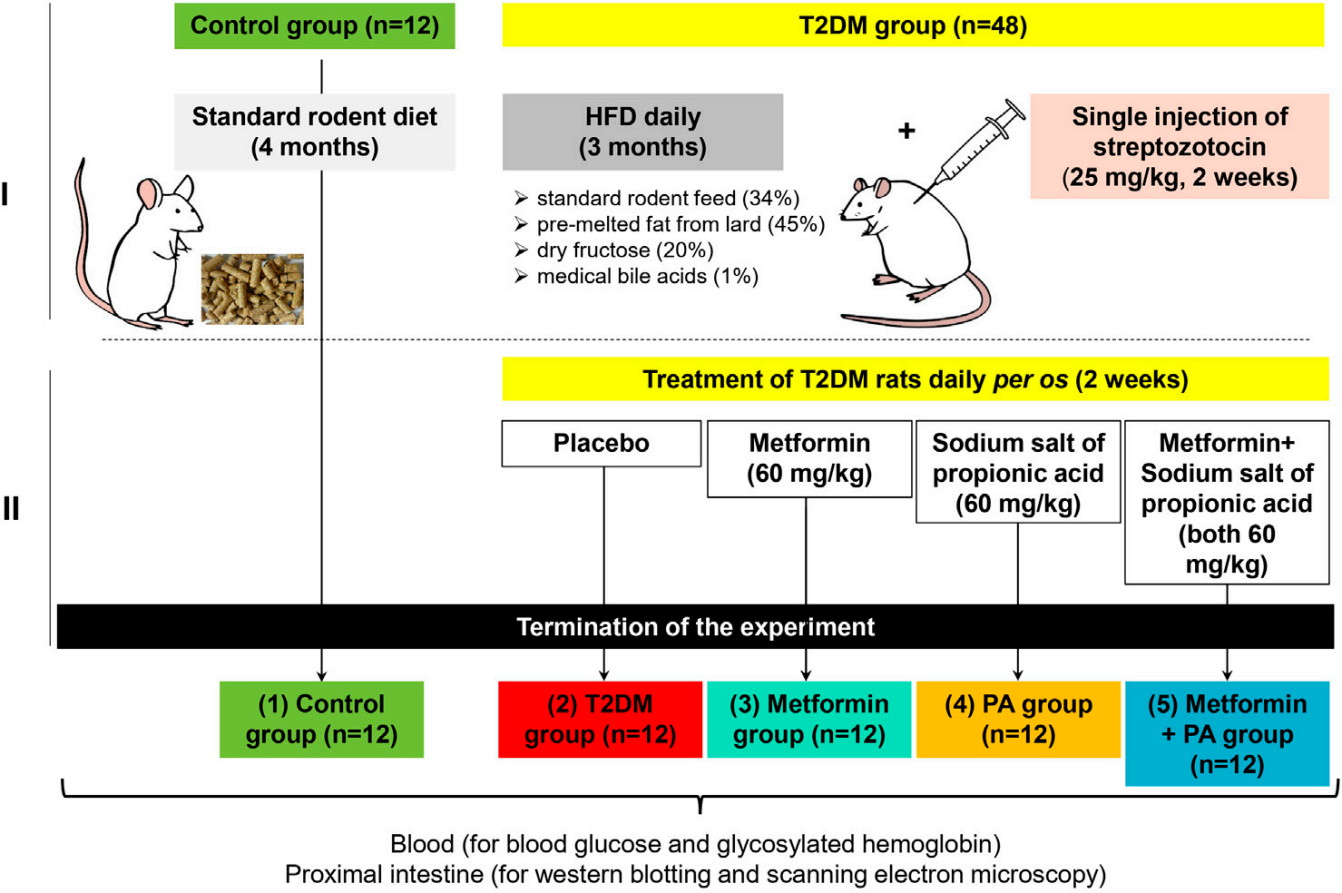
The general experimental scheme describing the design of the animal experiment and timescale. In the first part of the experiment **(A)**, animals were randomly allocated to 2 groups: 1) normal healthy rats on the standard rodent diet–the control group (n = 12); 2) the group with experimentally induced type 2 diabetes mellitus (n = 48). T2DM was developed after 3 months of high-fat diet with the following single injection of streptozotocin at a dose of 25 mg/kg of body weight (Sigma, United States). After confirmation of the development of stable hyperglycemia, in the second part of the experiment all animals with T2DM were randomly subdivided into additional treatment groups (n = 12) as follow **(B)**: 2) the group with experimentally induced T2DM; 3) the group of rats that received orally anti-hyperglycemic drug metformin (GLUKOFAGE, Merck Sante, France) at a dose of 60 mg/kg of b.w. for 14 days on the background of T2DM; 4) the group that received orally sodium salt of propionic acid (PROPICUM^®^, Flexopharm Brain GmbH & Co., Germany) at a dose of 60 mg/kg of b.w. for 14 days on the background of T2DM; 5) the group of T2DM rats that received concurrently metformin and sodium salt of propionic acid (both 60 mg/kg of b.w.) per os, for 14 days. After 2-week treatment with metformin and PA, all animals fasted for 12 h, then the blood samples were collected from the retro-orbital venous plexus under diethyl ether anesthesia. Then, rats were sacrificed, and samples of 1 cm of proximal intestine with the focus on the duodenum (the area of 3 cm distally) were collected for further western blot analysis and scanning electron microscopy (SEM).

The general experimental scheme describing the design of the animal experiment is shown in the Figure 1. In the first part of the experiment (Figure 1A), animals were randomly allocated to 2 groups: 1) normal healthy rats representing the control group (n = 12); 2) the group with experimentally induced type 2 diabetes mellitus (n = 48). The selection of animals to form experimental groups was performed by the method of “random numbers.” T2DM was induced by a 3-month high-fat diet (HFD) followed by an injection of a low dose of streptozotocin (STZ, Sigma, United States). Briefly, HFD mixture consisted of standard rodent feed (34%), pre-melted fat from lard (45%), medical bile acids (1%), and dry fructose (20%) as described previously (Natrus et al., 2019). STZ (25 mg/kg of b.w.) (Xiang et al., 2010; Hou et al., 2012) was injected once intraperitoneally after 3-month HFD. After confirmation of the development of stable hyperglycemia, all experimental animals with T2DM were randomly subdivided into additional treatment groups (12 rats/group) as follow (Figure 1B): 2) the group with experimentally induced T2DM; 3) the group of rats that received orally anti-hyperglycemic drug metformin (GLUKOFAGE, Merck Sante, France) at a dose of 60 mg/kg of b.w. (Alzamendi et al., 2012; Naik et al., 2021). for 14 days; 4) the group that received orally sodium salt of propionic acid (PROPICUM^®^, Flexopharm Brain GmbH & Co., Germany) at a dose of 60 mg/kg of b.w. (Zhou et al., 2021; Natrus et al., 2022). For 14 days; 5) the group of T2DM rats that received concurrently metformin and sodium salt of propionic acid (both 60 mg/kg of b.w.) *per os*, for 14 days.

### 2.2 Blood and tissue sampling

After the completion of 2-week treatment with metformin and PA, all animals fasted for 12 h, then the blood samples were collected from the retro-orbital venous plexus as described here (Sharma et al., 2014) under diethyl ether anesthesia. After blood collection, rats were sacrificed by cervical decapitation under deep anesthesia, and samples of 1 cm of small intestine with the focus on the duodenum (the area of 3 cm distally) were rapidly dissected for further western blot analysis and scanning electron microscopy (SEM).

### 2.3 Measurement of blood glucose and glycosylated hemoglobin

Blood glucose level was determined fasting in the morning using the One Touch Select biosensor (“Life Scan,” United States).

Blood glycosylated hemoglobin (HbA1c) content was measured using a standard kit «Hemoglobin A1C-Direct» (HbA1C-DIR, #31047, Bio Systems, Spain). Samples of 50 µL of whole blood, collected in the tube with EDTA, were mixed with 5 mL of distilled water, and then the mixture was applied on a microcolumn provided in the commercial kit. Filling buffer was used to elute the HbA1c fraction according to the manufacturer’s protocol. The optical density of collected eluates was measured spectrophotometrically at the 415 nm wavelength. The optical density of the hemolysed samples containing total hemoglobin was also determined, and the results were represented as a percentage (%) of the HbA1c fraction to the total hemoglobin content.

### 2.4 Electron microscopy

The samples of rat small intestine were fixed in 2.5% glutaraldehyde in Millonig’s phosphate buffer (рН 7.4) for 4 h, then transferred to 1% osmium tetroxide for 1 h. Fixed samples were poured into a mixture of epon-araldite (Sigma, United States) with step-by-step dehydration in ethanol and acetone. Ultrathin sections were contrasted with 2% uranyl acetate solution and lead citrate (TOV SPE “ALFARUS,” Ukraine), then examined with a scanning electron microscope Tescan Mira 3 LMU (TESCAN GROUP, Czech Republic) in a STEM mode (parameters: SEM HV 10.0kV, WB 3.93–3.97, Det TE). Morphometric measurement was carried out using Carl Zeiss software (AxioVision SE64 Rel.4.9.1), taking into account the approach published by Lam et al. (2021).

### 2.5 Protein analysis by western blotting

Protein levels of Bax, Bcl-X, caspase-3, and Ki67 were determined by western blot analysis. Total protein lysates from small intestine samples (0.1 g) were prepared using a standard protocol with a RIPA buffer (20 mM Tris-HCl, pH 7.5; 150 mM NaCl; 1% Triton X-100; 1 mM EGTA; 0.1% SDS; 1% sodium deoxycholate; 10 mM sodium pyrophosphate, all reagents were purchased from Sigma, United States) in the presence of protease inhibitor cocktail (Sigma, United States). Samples containing 50 μg of protein per track were subjected to 7%–15% SDS-PAGE (depending on the molecular weight of target protein). Resolved proteins were transferred to nitrocellulose membrane (0.45. µm, Merck Millipore, United States) in Tris-glycine buffer (25 mM Tris; 192 mM glycine; 20% methanol; pH 8.3) for 1 h. After 1-h blocking with 5% non-fat milk in phosphate-buffered saline plus 0.05% Tween-20 (PBST), membranes were incubated overnight at +4°C with primary antibodies against Bax (1:500, #МА5-14003, Invitrogen, United States), Bcl-X (1:1,000, #PA5-21676, Invitrogen, United States), caspase-3 p17 (1:250, sc-373730, Santa Cruz, United States), Ki67 (1:1,000, #MA5-14520, Invitrogen, United States) and β-actin (1:5,000, A3854, Sigma-Aldrich, United States) in PBST and 5% non-fat milk. After extensive washing, membranes were incubated with HRP-conjugated secondary antibodies for 1 h at a room temperature: anti-rabbit IgG (1:4,000, #A0545, Sigma-Aldrich, United States) or anti-mouse IgG (1:5,000, #ab97057, Abcam, United Kingdom). To visualize protein bands, an enhanced chemiluminescence was performed with p-coumaric acid (Sigma-Aldrich, United States) and luminol (Sigma-Aldrich, United States). The relative levels of Bax, caspase-3, and Ki67 were normalized to β-actin, and quantified with the software “Gel-Pro Analyzer32” (v3.1) as a fold change compared with the control level of a target protein.

### 2.6 Statistics

Data are presented as means ± S.E.M. All data were tested for significance using unpaired Student’s t-test or ANOVA. *p* < 0.05 was considered to be statistically significant. Statistical analysis was performed using “IBM SPSS Statistics for Windows, version 23” (SPSS Inc., United States) or StatPlus (AnalystSoft Inc., United States) (for electron microscopy measurements).

## 3 Results

### 3.1 Changes in animal body parameters, blood glucose and glycosylated hemoglobin levels of diabetic rats after metformin and PA administration

To verify the rat T2DM experimental model, we monitored a range of common characteristics including animal body parameters (final weight and waist), glucose and glycosylated hemoglobin levels in the serum of animals. After 3-month HFD and 2 weeks of treatment, the body weight and waist of T2DM rats were higher by 56% and 32% respectively compared with control animals (Table 1). Administration of metformin and PA, as well as their combination had no significant effect on body weight and waist compared with the T2DM group. However, the body weight in the metformin, PA and combined treatment groups was still higher by 47%, 44%, and 39% respectively compared with the control animals. The similar changes in the waist were also observed–this parameter was higher in the groups with metformin (by 27%), PA (by 25%) and their combination (by 22%) vs. control rats.

**TABLE 1:** Animal body parameters, blood glucose and HbA1c levels after metformin and propionic acid treatment on the background of type 2 diabetes.

Group Parameter	Control	T2DM	Metformin treatment	PA treatment	Metformin + PA treatment
Final weight, g	198.5 ± 8.4	308.8 ± 22.6*	291.1 ± 18.2*	285.5 ± 10.5*	275.1 ± 13.4*
Waist, cm	13.0 ± 0.8	17.2 ± 0.9*	16.5 ± 0.4*	16.3 ± 0.5*	15.8 ± 0.9*
Blood glucose, mmol/L	4.7 ± 0.4	12.6 ± 0.7*	13.5 ± 0.9*	13.2 ± 0.6*	11.8 ± 1.1*
Blood HbA1c (%)	5.5 ± 0.6	8.9 ± 0.9*	8.6 ± 0.8*	10.8 ± 0.5*^, &, #^	9.5 ± 0.7*

Note: Values are given as mean ± S.E.M. (n = 12); *p < 0.05 vs. control, ^&^ *p* < 0.05 vs. T2DM, ^#^ *p* < 0.05 vs. metformin administration.

As it is shown at Table 1, fasting blood glucose level in the T2DM group (12.6 ± 0.7 mmol/L) was significantly elevated (by 2.7 times) compared with the control group (4.7 ± 0.4 mmol/L). There was no statistically significant effect on glucose level after metformin (13.5 ± 0.9 mmol/L), PA action (13.2 ± 0.6 mmol/L) and their combined administration (11.8 ± 1.1 mmol/L) compared with the T2DM animals. To further characterize the state of hyperglycemia, we assessed the HbA1c level and found the similar pattern of changes within the experimental groups as it was observed for the glucose level. In particular, we observed the level of blood HbA1c 1.62 times higher in T2DM rats and 1.56 times higher in the metformin group vs. control. Interestingly, it should be noted that PA administration had a slight increasing effect on the HbA1c content not only compared with the control group (2-fold), but also in comparison with the T2DM rats (1.21-fold) and the metformin group (1.26-fold). There was no statistically significant difference in the level of HbA1c between the groups with a combination of metformin and PA and groups with separate administration of these drugs; however, it still remained 1.73-fold higher than in control animals.

Thus, based on the significant increase in the weight, waist, glucose and HbA1c serum levels, we confirmed the development of obesity, hyperglycemia and experimentally induced T2DM. Administration of PA and metformin at doses of 60 mg/kg for 2 weeks had almost no pronounced effect on these parameters on the background of T2DM.

### 3.2 Characterization of ultrastructural parameters of mitochondria in the small intestine of rats with T2DM after metformin and PA administration

**FIGURE 2:**
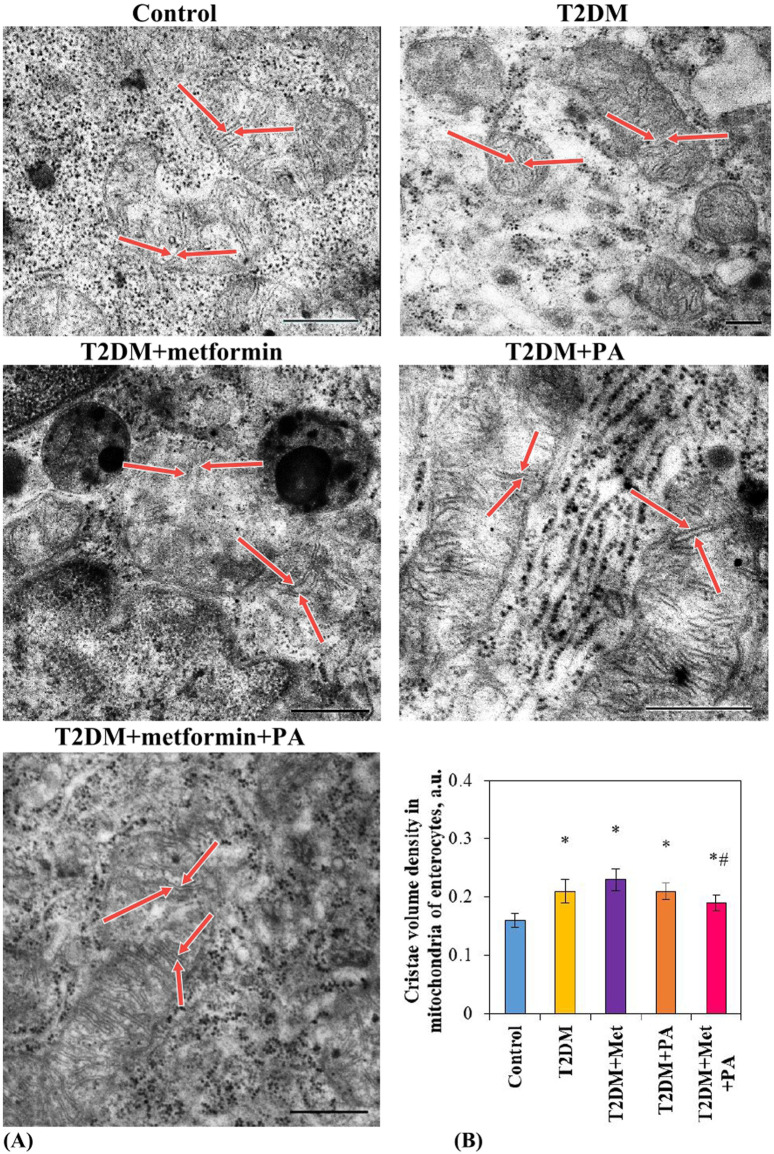
Ultrastructural changes of mitochondria in enterocytes of T2DM rats after metformin and PA administration. Representative images of mitochondria in enterocytes **(A)** of 5 experimental groups: 1) control rats; 2) rats with T2DM; 3) rats with T2DM treated with metformin; 4) rats with T2DM treated with PA; 5) rats with T2DM treated with a combination of metformin and PA, were obtained by scanning electron microscopy (n *=* 10 enterocytes for each group). Red arrows indicate cristae in mitochondria. ImageJ software was used to calculate the mitochondria structural parameter–cristae volume density **(B)** in each cell based on the electronic microscopic microphotographs. Values are given as mean ± S.E.M. Scale bar: 500 nm; *p < 0.05 vs. control, ^#^ *p* < 0.05 vs. metformin administration.

Electron microscopic examination (Figure 2A) revealed a slight increase in the size of cristae (red arrows) and the cristae volume density in mitochondria of enterocytes in the T2DM group (0.21 ± 0.02 a.u.) compared vs. the control animals (0.16 ± 0.01 a.u). As it was presented in Figure 2B, morphometric analysis also showed a tendency to an increased cristae volume density after metformin (0.23 ± 0.02 a.u.) and PA administration (0.21 ± 0.01 a.u.), however, there was no statistically significant difference between these correction groups and T2DM. In the group with a combination of drugs, a tendency to normalization of the ultrastructural parameter of mitochondria was observed (0.19 ± 0.01 a.u.), and a decrease compared to the metformin group was noted (*p* = 0.04). Moreover, after metformin and PA combination, no significant difference in the ultrastructure of mitochondria in enterocytes detected by visual assessment was found compared to the control animals. It should be noted, that in all studied groups, phagosomes and, in less extent, residual bodies were found in enterocytes. Their ultrastructural characteristics were quite polymorphic, the size and the electron density had significant differences between cells, even within the same group. Interestingly, enterocytes with an accumulation of phagosomes/lysosomes were observed after a separate use of metformin and PA.

### 3.3 The ratio of apoptotic and proliferative markers in the small intestine of rats with T2DM after metformin and propionic acid treatment

To characterize the direction of apoptotic and proliferative processes, we measured the protein levels of the major players–Bax, Bcl-X, caspase-3 and Ki67 in the duodenum of diabetic rats and after administration of studied compounds.

**FIGURE 3:**
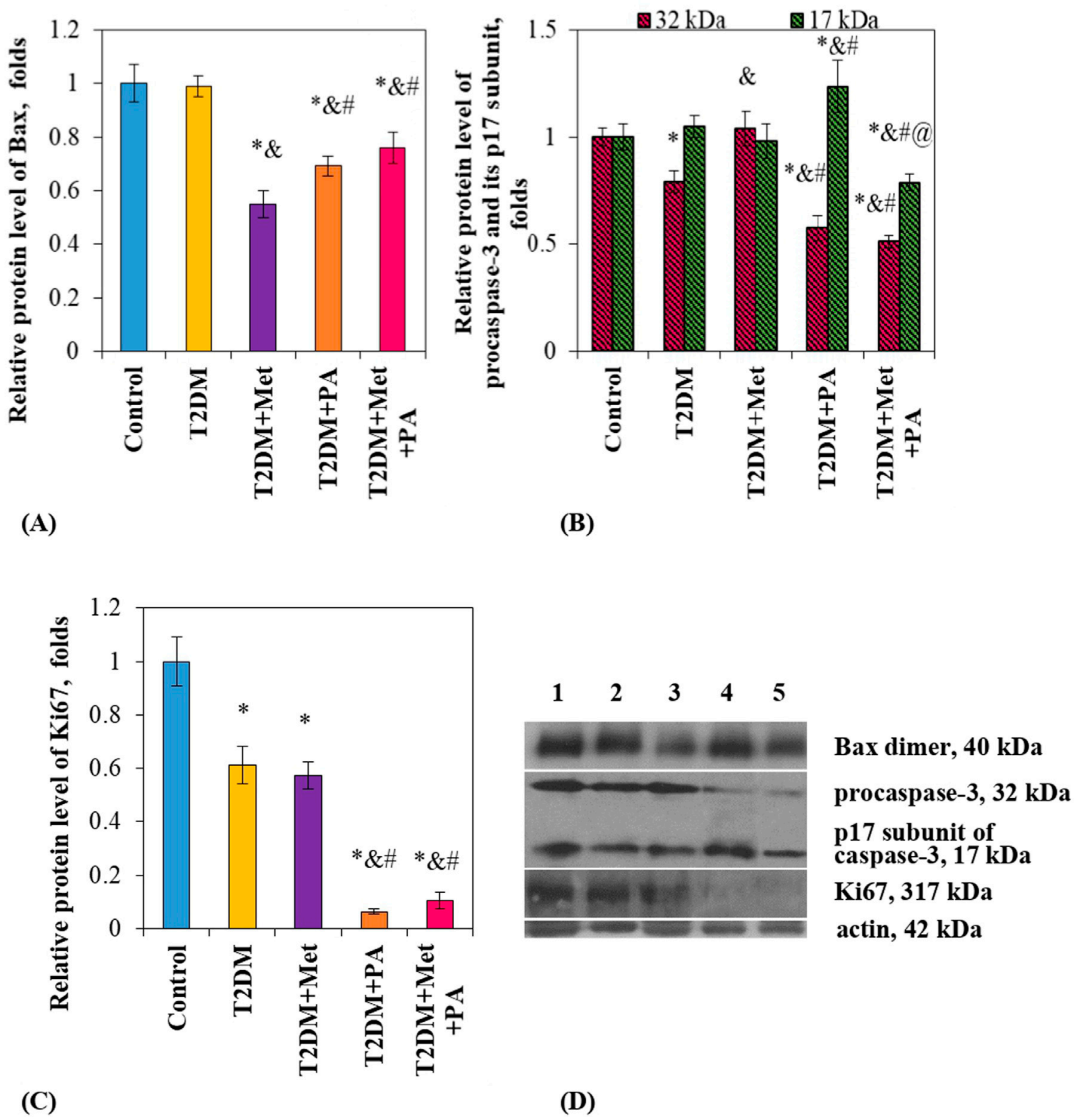
Metformin and PA action on the apoptotic and proliferative markers in the small intestine. Immunoblotting analysis of Bax, caspase-3 and Ki67 was performed in rat small intestine of 5 experimental groups: 1) control rats; 2) rats with T2DM; 3) rats with T2DM treated with metformin; 4) rats with T2DM treated with PA; 5) rats with T2DM treated with a combination of metformin and PA. The bar graphs of Bax **(A)**, procaspase-3 (32 kDa) and p17 subunit (17 kDa) of caspase-3 **(B)**, Ki67 **(C)** are presented as mean ± S.E.M. (n = 6). Representative immunoblots are shown **(D)** and quantified using β-actin as a loading control for intestinal lysates; *p < 0.05 vs. control, ^&^ *p* < 0.05 vs. T2DM, ^#^ *p* < 0.05 vs. metformin administration, ^@^ *p* < 0.05 vs. PA administration.

First, we started from the determination of the level of proapoptotic Bcl-2-associated X protein (Bax), one of the essential gateways to cell death via mitochondria (Peña-Blanco and García-Sáez, 2018). Interestingly, the Bax content in the small intestine of diabetic rats was unchanged compared vs. control animals (Figures 3A, D). Studied compounds led to a decrease in the Bax level in different extent–by 45% after metformin, by 21% after PA, and by 24% after a combination of them. Unfortunately, we were not able to identify antiapoptotic Bcl-X protein in the duodenum by western blotting, suggesting that chosen Bcl-X antibody did not detect the small amount of this protein in the intestinal lysates.

Second, we determined the level of another apoptotic marker–caspase-3. Caspase-3 is linked directly to the Bax/Bcl-X family member proteins, since it cleaves Bcl-2 and Bcl-X destroying their antiapoptotic function. Our antibody allowed to detect two forms of caspase-3: procaspase-3, which exists in cells as an inactive 32 kDa prozyme, and its cleaved 17 kDa subunit. In the T2DM group, there was no changes detected in the p17 subunit level, while procaspase-3 content slightly declined compared to the control by 21% (Figures 3B, D). Interestingly, metformin did not affect the levels nor procaspase-3 neither p17 subunit compared vs. control. In contrast, PA exerted multidirectional effects: while inactive procaspase-3 content was declined by 42% vs. control), its active subunit p17 was elevated by 23.7% vs. control. Finally, a combination of these drugs did not change procaspase-3 content compared to the PA group, while there was a decrease in the p17 subunit level compared to all groups: vs. PA–by 36.3%, vs. metformin–by 19.6%, vs. T2DM–by 25%, and vs. control animals–by 21.2% (Figures 3B, D).

Finally, we assessed the intestinal proliferative potential based on the Ki67 level. We found an interesting observation that both diabetes and treatment with different compounds led to a decrease in Ki67 content by different extent compared to the control rats: by 38.8% in the T2DM rats, by 42.7% in the metformin group, by 93.7% in the PA group, and by 89.5% in the metformin + PA group, suggesting that PA almost fully inhibited the proliferation in the small intestine (Figures 3C, D).

## 4 Discussion

The focus for pharmacological approaches to counteract diabetes-induced and metformin-related gastrointestinal complications should be directed to the two critically important processes for intestinal renewal–programmed cell death and cell proliferation. Apoptosis is the cellular process that ensure the normal functioning of the intestinal epithelium. At the same time, under pathological conditions the excessive cell death may lead to the chronic inflammation (Subramanian et al., 2020). In turn, a strict equilibrium between cell proliferation in the crypt and cell shedding from the small intestinal villus tip maintains intestinal epithelial homeostasis (Negroni et al., 2015). Considering that there is a general lack of studies dedicated to comparative effectiveness of metformin monotherapy and its combination with the supportive therapy for GI complications, in this study, we explored the impact of metformin and PA on apoptosis and proliferation in the small intestine.

Since unresolved endoplasmic reticulum (ER) stress in intestinal cells may lead to the development of intestinal inflammation and, as a result, enhance programmed cell death (Eugene et al., 2020), our hypothesis was that diabetes-associated perturbation in intestinal homeostasis may be linked to an impaired balance between apoptotic and proliferative processes. In our previous research on diabetes-related influence on enterocytes, we have studied the UPR state in the small intestine and showed the ER stress and UPR activation in enterocytes of diabetic rats (Natrus et al., 2024). Therefore, it was not a surprise that we observed an increase in the cristae volume and mitochondria swelling, which may indicate the damage to the energy apparatus of enterocytes in T2DM animals. Our data also were in line with the study that showed a strong link between mitochondrial dysfunction and the pathology of T2DM that may lead to leptin and insulin resistance (Rocha et al., 2016).

Based on the data that cytochrome c released from mitochondria may activate proapoptotic molecules including caspase-9-caspase-3 cascade and, moreover, caspase-3 is participated in the process of TNFα-induced intestinal epithelial cell shedding and further disruption of the intestinal barrier integrity (Williams et al., 2013), we determined the levels of proapoptotic factors. Intriguingly, there was no effect on the Bax and caspase-3 p17 subunit levels, showing that there was no diabetes-induced activation of apoptotic pathways in the duodenum. This led us to the suggestion that hyperglycemia and hyperlipidemia caused mainly inflammatory damage and direct destruction of intestinal cells than via necrosis. At the same time, we observed reduced Ki67 content, suggesting a decrease in the proliferative potential. In turn, reduced proliferative potential in T2DM rats may indicate insufficient and delayed intestinal regeneration, which may possibly explain the enteropathy and dyspeptic phenomena in T2DM patients (Meldgaard et al., 2018).

Interestingly, oral metformin bioavailability is 50%–60%, and depends on the stomach and intestinal peristalsis as well as may decrease after high-fat meal consumption or under pathological conditions (Graham et al., 2011). Thus, we assumed that T2DM-related intestinal impairments may also decrease metformin bioavailability thus diminishing its therapeutic action. Today we observe a renewed interest to the gut as a target organ for metformin. Interestingly, in the context of gut, metformin enhances anaerobic glucose metabolism in enterocytes, resulting in decreased glucose uptake and increased lactate delivery to the liver (Rena et al., 2017). In turn, increased local concentrations of lactate may contribute to the development of gastrointestinal symptoms associated with metformin intolerance (McCreight et al., 2016).

Additionally, it was reported that metformin improves mitochondrial function in peripheral blood mononuclear cells from type 2 diabetic patients (de Marañón et al., 2022). In contrast, we showed that treatment with metformin led to a maximum increase in the cristae volume of enterocytes. At first glance, this increase may be a sign of swelling, therefore, unfavorable for the organelle functioning. However, there was another possible explanation. It was shown that metformin concentrated within mitochondria up to 1,000 times higher compared to extracellular environment (Bridges et al., 2014) possibly leading to the cristae enlargement. Simultaneous critical decrease in the Bax content, one of the core regulators of the intrinsic pathway of apoptosis, after metformin monotherapy may indicate the inhibition of membrane pore formation on the outer membrane of mitochondria (Subburaj et al., 2015) and, as a result, impaired mitochondria-dependent programmed cell death and mitochondrial fusion process in small intestine (Hoppins et al., 2011). In any case, we showed metformin-caused decrease in Bax without any influence on the procaspase-3 and p17 subunit levels. Although the role of apoptosis in the structural integrity of the gut is still controversial, the question of the advisability of suppressing of Bax-mediated mitoptosis in enterocytes arises, since these cells should be regularly renewed for normal functioning. A decrease in Bax-mediated apoptosis occurred concurrently with the reduced Ki67 content. Observed slowdown of proliferative processes after metformin therapy was expected and may be explained by the previously reported ability of metformin to reduce the risk of cancer both in diabetic and nondiabetic populations (Kasznicki et al., 2014). This antiproliferative and anticancer action may be rather attractive property of metformin. However, if we consider the balance between apoptosis and proliferation as adaptive process that ensure regular tissue renewal, then we can assume the undesirable suppression of reparative processes in the small intestine after metformin monotherapy.

Since large metagenomic study described T2DM-associated gut microbial dysbiosis and a decrease in the number of bacteria producing butyrate and propionate on the background of increased number of opportunistic microorganisms (Forslund et al., 2015), we suggest that PA may be a perspective candidate to modulate the key molecular/cellular events that were impaired during long-term T2DM treatment with metformin. However, we could not consider propionate exclusively from a positive point of view, since there is a pool of data highlighted PA as a potential metabolic disruptor. Authors in this study (Adler et al., 2021) discovered that oral consumption of PA led to inappropriate activation of the insulin counterregulatory hormonal network that is especially needs attention in the context of diabetes studies. Moreover, considering that PA and propionate-containing diet supplements may be used as an effective therapy for neurodegenerative diseases, the question about propionate toxicity that lead to a number of side effects (Witters et al., 2016; Lobzhanidze et al., 2020; Lagod et al., 2024) remains debatable. Therefore, the relevance of our work raised from two multidirectional aspects: first, the question of how exogenous PA can affect glucose metabolism is still not fully understood, and second, whether PA may be a unifying therapy for diabetes-induced neuropathies and gastrointestinal complications without considerable side effects. Therefore, future studies are needed to determine which effect of PA consumption on the intestine would prevail: positive or negative.

Since cell death in the intestinal epithelium may be regulated by gut microbiota (Becker et al., 2013) and their metabolites as SCFAs, we assumed that PA may influence cell death and proliferation processes. Despite reported beneficial effects of PA, its excess may be associated with human diseases involving mitochondrial dysfunction as propionic acidemia and autism spectrum disorders (Frye et al., 2016). However, in our case PA administration had no valuable effects on cristae volume density of enterocytes compared to the T2DM and metformin groups. The proposed explanation is that enteric microbiome metabolites such as PA may have both beneficial and toxic effects on mitochondrial function, depending on tissue type, concentration, exposure duration and microenvironment redox state (Frye et al., 2016). However, on the molecular level we observed unexpected changes in the patterns of proapoptotic and proliferative markers–on the background of dramatically decreased Ki67 level and moderately reduced Bax and procaspase-3 content, there was an elevation in active caspase-3 p17 subunit. Thus, the molecular mechanism of PA action on the small intestine is primarily mediated via enhanced caspase-3-dependent apoptosis with almost totally inhibited proliferation. Earlier, sodium propionate was shown to promote apoptosis and autophagy pathways through a peroxisome proliferator activated receptor gamma (PPAR-γ)-dependent mechanism suggesting that the PPAR-γ/SCFAs axis could be targeted for the glioblastoma management (Filippone et al., 2022). In addition, revious studies convincingly proved that SCFAs, including PA, are considered one of the effective oncoprotective drugs (Pham et al., 2021).

Finally, after a combination of metformin and PA, we observed positive changes at the cellular level: the swelling of mitochondria was decreased, their ultrastructural characteristics, size and electron density was more close to the control, than after metformin monotherapy. Interestingly, exactly the combination of PA and metformin exerted such effect, not PA monotherapy. One of the possible reasons for this effect may be partial normalization of apoptotic potential based on the Bax elevation compared vs. metformin monotherapy, which we consider extremely important for cells that are constantly involved in the transport of substances from the blood to the small intestine.

Thus, we obtained completely new experimental data on changes in mitochondria of enterocytes in metformin-treated rats on the background of T2DM and evaluated the advisability of additional PA treatment as a supportive GI therapy. The following limitations need to be considered when interpreting the results. First, we cannot measure Bcl-X level by western blotting, possibly due to its extremely small amount in intestinal lysates that cannot be detected by chosen Bcl-X antibody in our working protocol. Second, advisability of PA supplementation for the improvement of diabetes-induced intestinal complications concurrently with metformin still remain debatable due to its ambiguous effects. Therefore, further studies are necessary to accurately identify the ratio between possible favorable and side effects after treatment with a combination of metformin and PA. However, despite these limitations, we assume that PA, as a one of the major SCFAs, is safe for intestine, and obtained data may be taken into account during the development and evaluation of new effective treatment strategies for diabetes-induced intestinal complications on the background of long-term metformin treatment.

## 5 Conclusion

This study compares the influence of two commonly used compounds–metformin and PA as well as the effectiveness of their combination on the T2DM-induced intestinal complications. We observed T2DM-induced profound changes in small intestine: impaired ultrastructure of mitochondria, increased cristae volume, and decreased level of proliferative marker Ki67, while the apoptotic ones were unchanged. Metformin and PA monotherapies did not change the cristae volume density compared with diabetic animals, however, after their combination a tendency to normalization of the ultrastructural parameters of mitochondria was observed. The main finding of this study is the identification of specific effects of metformin and PA on apoptotic processes in the duodenum: while metformin treatment inhibited apoptosis mostly via Bax declining, PA mainly acted via caspase-3-dependent mechanism. Diabetes-induced inhibition of the intestinal proliferation was deepen after treatment with both PA and metformin that requires further investigation. However, these data may be taken into account during the development of new supportive treatment strategies for diabetes-induced intestinal disturbances on the background of metformin treatment. PA supplementation for the improvement of diabetes-induced gastrointestinal complications concurrently with metformin may be consider as a perspective supportive therapy.

## Data Availability

The raw data supporting the conclusions of this article will be made available by the authors, without undue reservation.
