# Supplementary material for: Apoptotic and proliferative processes in the small intestine of rats with type 2 diabetes mellitus after metformin and propionic acid treatment
Source: Front Pharmacol. 2024 Oct 16;15:1477793. doi: 10.3389/fphar.2024.1477793 (PMC11521949; doi:10.3389/fphar.2024.1477793)
Supplement: Supplementary file 1 [file DataSheet1.PDF]

Supplementary Information file 1
Initial set of Western blot experiments
(full-length, original, unprocessed versions of blots)

Raw data from Western blot experiments using Bax antibody
(Fig. 3 in the manuscript)

Immunoblotting was conducted with primary antibody against Bax (1:500, #MA5-14003, Invitrogen, USA), 10 % PAAG, 50 µg of protein per track. Samples are labelled (according to the Table 1) above the images.

Table 1 Sample labelling

Gel 1 (Bax)	Gel 2 (Bax)
1 – marker;	1 – marker;
2 – Control (31);	2 – Control (33);
3 – DM2 (52);	3 – DM2 (54);
4 – DM2 + met. (41);	4 – DM2 + met. (45);
5 – DM2 + prop. (62);	5 – DM2 + prop. (62);
6 – DM2 + comb. (71);	6 – DM2 + comb. (74);
7 – Control (32);	7 – Control (33);
8 – DM2 (53);	8 – DM2 (52);
9 – DM2 + met. (43);	9 – DM2 + prop. (62);
10 – DM2 + prop. (62).	10 – DM2 + comb. (75).

Notes:
marker – protein ladder;
Control – the control group;
DM2 – the group with experimentally induced type 2 diabetes mellitus (T2DM);
DM2 + met. – the T2DM group that received anti-hyperglycemic agent metformin;
DM2 + prop. – the T2DM group that received sodium salt of propionic acid (PA);
DM2 + comb. – the T2DM group that received concurrently metformin and PA.

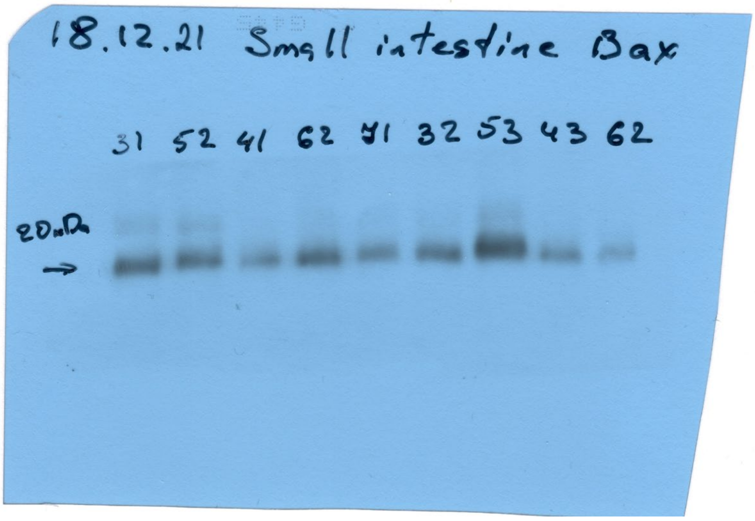

1 gel | 1 min exposition

1 2 3 4 5 6 7 8 9 10

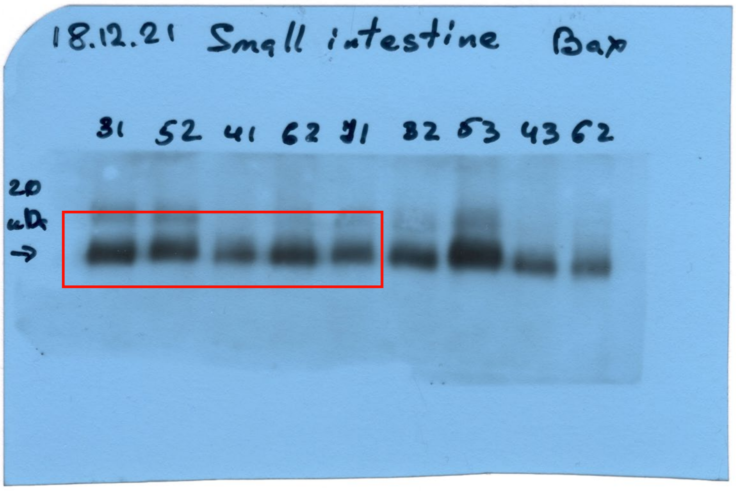

1 gel 3 min exposition

1 2 3 4 5 6 7 8 9 10

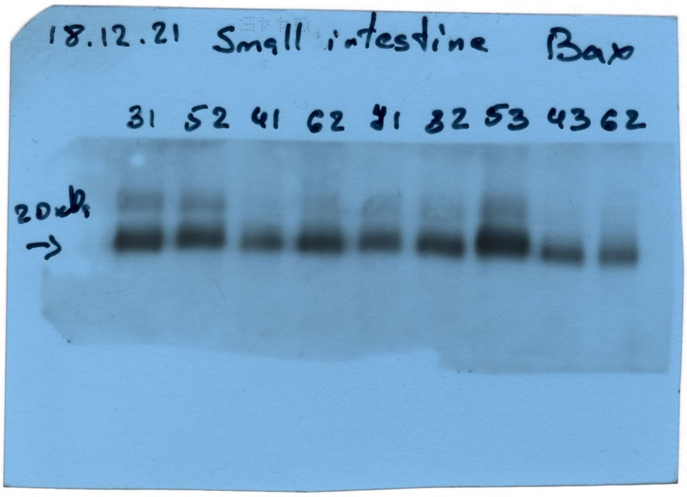

1 gel 5 min exposition

1 2 3 4 5 6 7 8 9 10

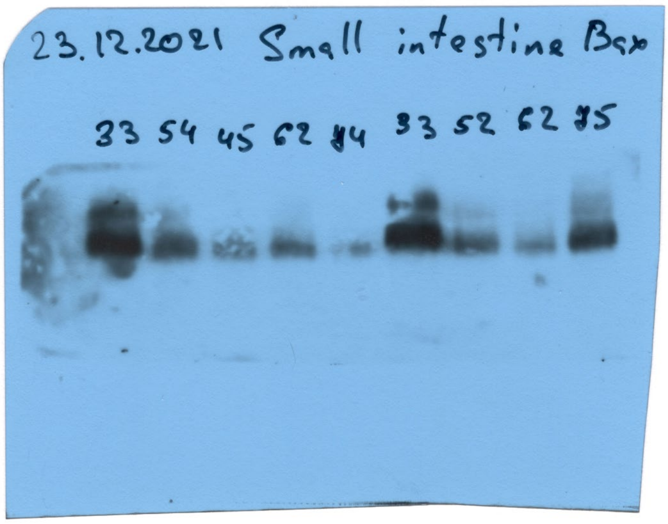

2 gel 5 min exposition

1 2 3 4 5 6 7 8 9 10

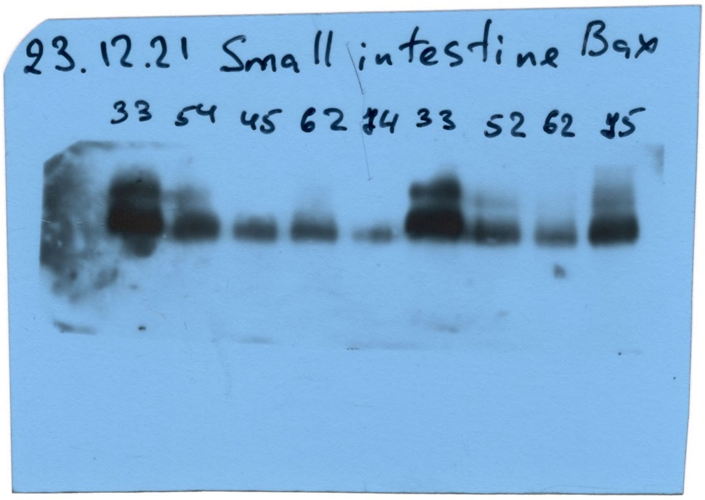

2 gel 7 min exposition

1 2 3 4 5 6 7 8 9 10

Raw data from Western blot experiments using β-actin antibodies
(Fig. 3 in the manuscript)

Immunoblotting was conducted with primary antibody against β-actin (1:5000, A3854, Sigma-Aldrich, USA), 10 % PAAG, 50 µg of protein per track. Samples are labelled (according to the Table 2) above the images.

Table 2 Sample labelling

Gel 1 (β-actin)	Gel 2 (β-actin)
1 – marker;	1 – marker;
2 – DM2 + met. (45);	2 – Control (31);
3 – DM2 + prop. (62);	3 – DM2 (52);
4 – Control (33);	4 – DM2 + met. (41);
5 – DM2 (54);	5 – DM2 + prop. (62);
6 – DM2 + comb. (74);	6 – DM2 + comb. (71);
7 – DM2 (52);	7 – Control (32);
8 – DM2 + met. (45);	8 – DM2 (53);
9 – DM2 + prop. (62);	9 – DM2 + met. (43);
10 – DM2 + comb. (75).	10 – DM2 + prop. (62).

Notes:
marker – protein ladder;
Control – the control group;
DM2 – the group with experimentally induced type 2 diabetes mellitus (T2DM);
DM2 + met. – the T2DM group that received anti-hyperglycemic agent metformin;
DM2 + prop. – the T2DM group that received sodium salt of propionic acid (PA);
DM2 + comb. – the T2DM group that received concurrently metformin and PA.

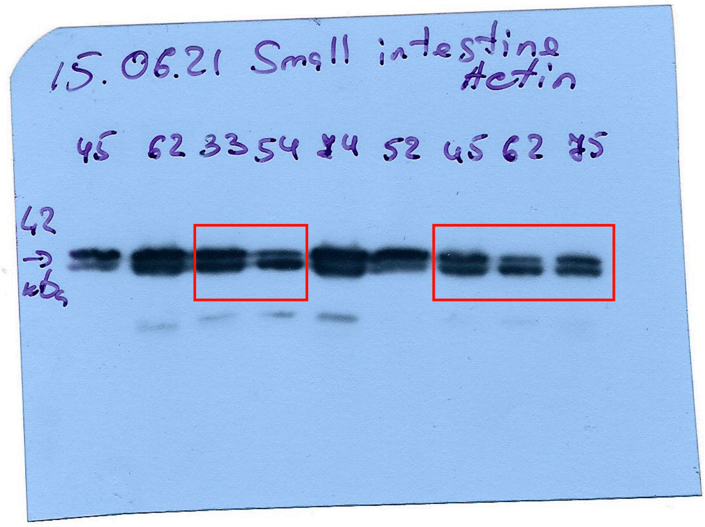

1 gel

30 sec exposition

1 2 3 4 5 6 7 8 9 10

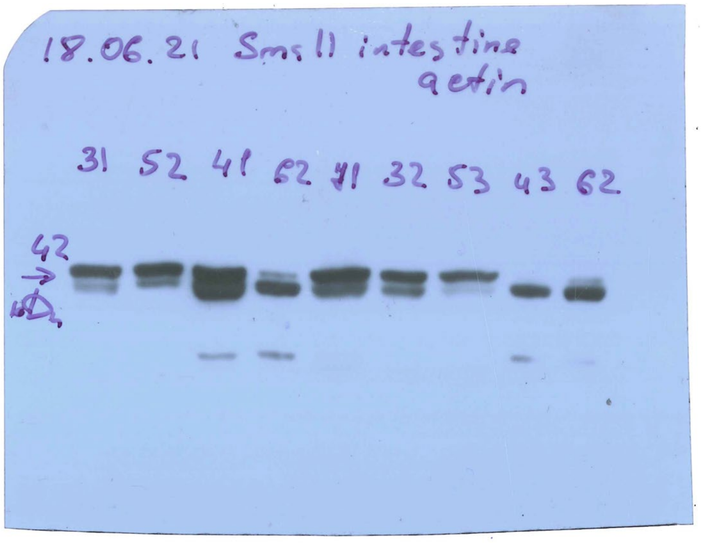

2 gel

30 sec exposition

1 2 3 4 5 6 7 8 9 10

Raw data from Western blot experiments using Ki67 antibody (Fig. 3 in the manuscript)

Immunoblotting was conducted with primary antibody Ki67 (1:1000, #MA5-14520, Invitrogen, USA), 7.5% PAAG respectively, 50 µg of protein per track. Samples are labelled (according to the Table 3) above the images.

Table 3 Sample labelling

Gel 1 (Ki67)
1 – Control (31);
2 – DM2 (52);
3 – DM2 + prop. (62);
4 – DM2 + prop. (66);
5 – DM2 + comb. (71);
6 – DM2 + met. (41);
7 – DM2 + met. (43);
8 – DM2 + met. (45);
9 – DM2 + comb. (75).
10 – marker (M);

Notes:
marker – protein ladder;
Control – the control group;
DM2 – the group with experimentally induced type 2 diabetes mellitus (T2DM);
DM2 + met. – the T2DM group that received anti-hyperglycemic agent metformin;
DM2 + prop. – the T2DM group that received sodium salt of propionic acid (PA);
DM2 + comb. – the T2DM group that received concurrently metformin and PA.

31 52 62 66 71 41 43 45 75 M

1 gel 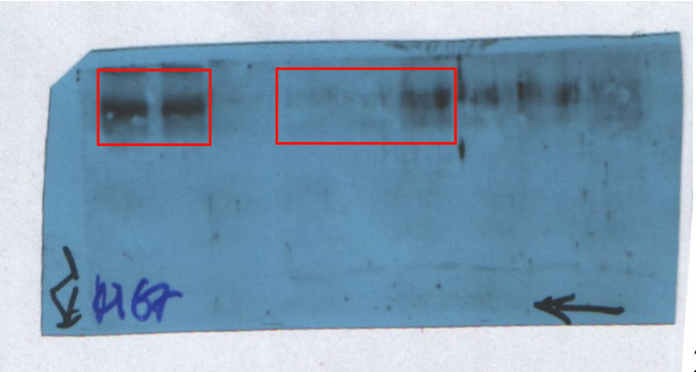 20 min exposition

1 2 3 4 5 6 7 8 9 10

Raw data from Western blot experiments using caspase-3 antibody (Fig. 3 in the manuscript)

Immunoblotting was conducted with primary antibody against caspase-3 p17 (1:250, sc-373730, Santa Cruz, USA), 15 % PAAG, 50 µg of protein per track. Samples are labelled (according to the Table 4) above the images.

Table 4 Sample labelling

Gel 1 (Ki67)
1 – marker;
2 – Control (31);
3 – DM2 (52);
4 – DM2 + met. (43);
5 – DM2 + met. (41);
6 – DM2 + comb. (74);
7 – Control (32);
8 – DM2 (53);
9 – DM2 + prop. (62);
10 – DM2 + prop. (66).

Notes:
marker – protein ladder;
Control – the control group;
DM2 – the group with experimentally induced type 2 diabetes mellitus (T2DM);
DM2 + met. – the T2DM group that received anti-hyperglycemic agent metformin;
DM2 + prop. – the T2DM group that received sodium salt of propionic acid (PA);
DM2 + comb. – the T2DM group that received concurrently metformin and PA.

M 31 52 43 41 74 32 53 62 66

1 gel 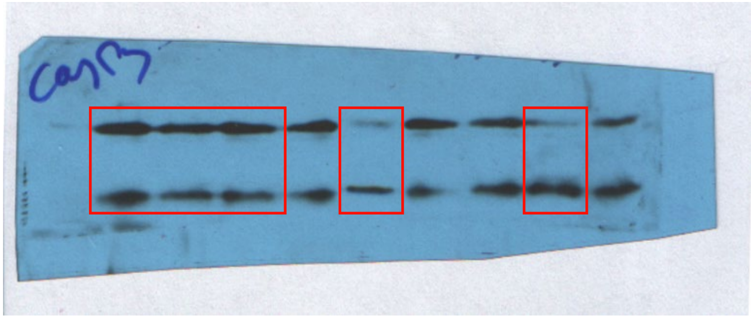 25 min exposition

1 2 3 4 5 6 7 8 9 10
